# Lectin affinity-based glycoproteome analysis of the developing xylem in poplar

**DOI:** 10.48130/FR-2022-0013

**Published:** 2022-10-27

**Authors:** Hao Cheng, Jinwen Liu, Meiqi Zhou, Yuxiang Cheng

**Affiliations:** 1 Key Laboratory of Tree Genetics and Breeding, Northeast Forestry University, Harbin 150040, China; 2 Zhejiang Provincial Key Laboratory for Water Environment and Marine Biological Resources Protection, Wenzhou University, Wenzhou 325035, China

**Keywords:** Glycoproteome, Xylem, Poplar, Lectin, Wood formation

## Abstract

Glycosylation is a significant post-translational modification of proteins, and some glycoproteins serve as players in plant cell wall synthesis and modification. Wood is a highly developed cell wall organization, and protein glycosylation as a regulatory mechanism may be involved in wood formation. Here, a lectin affinity-based glycoproteome was performed in stem developing xylem of poplar. After enrichment, trypsin digestion, LC-MS/MS analysis and peptide identification, we identified 154 glycoproteins from poplar developing xylem, which were classified into nine functional groups mainly including protein acting on carbohydrates, oxido-reductase, proteases, and protein kinases. Further, N- and/or O-glycosylation sites of the identified proteins were analyzed using bioinformatic tools, and deglycosylation experiments in the selected PtSOD and PtHAD proteins verified the reliability of the identified glycoproteins. Analysis of protein subcellular localization showed that a total of 63% of the identified glycoproteins were extracellular proteins or located in the plasma membrane. Poplar eFP and RT-qPCR data showed that a number of the genes encoding these glycoproteins such as laccase, peroxidase and cysteine protease, have highly preferential expression profiles in the developing xylem. Together with previously published research, most identified glycoproteins could be involved in wood cell wall synthesis and modification in poplar. Thus, our study provides some potential wood formation-related glycoproteins to be determined during tree stem development.

## INTRODUCTION

Plant cell walls are complex and dynamic structures mostly composed of diverse polysaccharides and cell wall proteins (CWPs)^[1]^. Plant primary cell walls (PCWs) are dynamically modified, reorganized, and loosened to allow for wall relaxation and cell expansion^[2]^. Polysaccharides of plant PCWs mainly contain pectin, hemicelluloses and cellulose. In addition, some cells of certain plant tissues develop thick secondary cell walls (SCWs). These specialized cells form SCWs inside the PCWs upon completion of cell expansion, which provide mechanical strength and water-conducting capabilities^[3,4]^. CWPs play key roles in cell wall formation and modification and adaptation to the environment^[5, 6]^. Wood is a highly developed cell wall organization, of which the fibers and vessels develop thick SCWs. Some glycosylated proteins have been proposed to function in wood formation via genetic evidence^[7]^.

Glycosylation is an important post-translational modification of proteins, affecting many protein functions and cellular activities. It is estimated that 50% of all proteins are glycosylated based on the fact that two-thirds of entries in the Swiss-Prot database were found to contain at least one *N*-glycosylation consensus (NXS/T)^[8,9]^. There are two main types of protein glycosylation: N-glycosylation and O-glycosylation in plants^[10]^. N-linked glycosylation is a common feature of plant proteins, particularly the CWPs that fulfill important roles in cell wall modification, sugar metabolism, signaling, and defense^[11]^. In addition, a structural role of hydroxyproline-rich O-glycoprotein extensins (EXTs) has been clearly assigned in maintaining the growing cell walls^[12]^. Therefore, it is necessary to identify the glycoproteins of tree stem xylem for the understanding of wood formation.

Lectin affinity chromatography (LAC) is a technique that uses different immobilized lectins to reversibly bind to specific sugar residues of the glycoproteins. Because of its specificity and rapidity, LAC has become a common tool for affinity purification of the glycoproteins^[13]^. Coupled with mass spectrometric (MS) technologies, a growing number of glycoproteome have been performed in different species. Over two decades, some glycoproteins have been identified as biomarkers or indicators of disease^[14−16]^. In plants, glycoproteome has been performed in tomato, Arabidopsis, rice, and cotton^[17−22]^. These studies indicate that the CWPs are a large number of glycoproteins. However, little is known about the identification of the glycosylated proteins in stem xylem in trees.

Poplar is a fast-growing tree with a large biomass accumulation in terrestrial ecosystems, extensively used in the pulp and paper industry, reforestation of land and bioenergy feedstocks. The objective of this study was to perform multiple lectin affinity-based glycoproteome of stem developing xylem in poplar. As a result, we identified 154 glycoproteins from the developing xylem of poplar. These glycoproteins were divided into nine functional groups, and 63% were located in the cell wall and the plasma membrane. In combination with the previously published research on functional reports, some glycoproteins are proposed to be involved in cell wall synthesis modification during wood formation. However, a large number of the genes encoding the identified glycoproteins are not still elucidated in function. Our study provides a significant foundation for further investigations into the potential roles of the identified glycoproteins in wood formation.

## RESULTS

### Multiple lectin affinity enrichment of glycoproteins in Populus developing xylem

**Figure 1:**
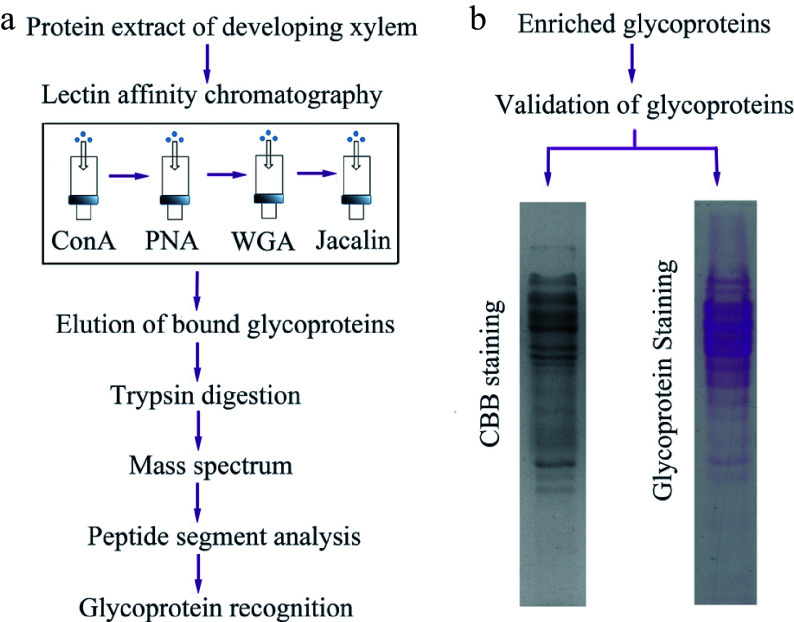
The workflow of glycoprotein enrichment and identification in poplar developing xylem. (a) Enrichment and recognition of the glycoproteins. Crude proteins were extracted from poplar developing xylem, and the glycoproteins were bound to ConA, PNA, WGA and jacalin lectin affinity columns. The eluted glycoproteins were digested by trypsin and the peptide segments were further recognized through mass spectrum analysis. (b) Validation of the eluted glycoproteins. The glycoproteins separated on sodium dodecyl sulfate-polyacrylamide gel electrophoresis (SDS-PAGE) gels were stained using Coomassie brilliant blue (CBB) staining and Pierce Glycoprotein Staining kit (detecting sugar moieties of the glycoproteins), respectively.

Total soluble proteins were extracted from developing xylem tissues of three-year-old poplar trees. The experiment was performed three times, and all extracts were mixed into total crude proteins. To enrich more numerous glycoproteins, we designed an affinity purification strategy, in which four kinds of plant lectins were integrated to enrich different types of glycoproteins (Fig. 1a). Concanavalin A (ConA) and wheat germ agglutinin (WGA) bind to the glycoproteins with mannosyl and glucosyl residues and N-acetyl-glucosamine and sialic acid, respectively^[23−27]^. Jacalin specifically binds galactosyl (β-1,3) N-acetylgalactosamine of O-linked glycoproteins, while peanut (PNA) can specifically recognize β-galactose^[28, 29]^. After total crude proteins flowed through the ConA-WGA-Jacalin-PNA affinity column, the captured proteins were eluted with the Elution Buffer containing 500 mM methyl D-glucopyranoside for Con A, 500 mM N-Acetyl-D-glucosamine for WGA, 500 mM N-Acetyl-D-galactosamine for PNA and 500 mM galactose for Jacalin, respectively. Afterwards, the eluted proteins were analyzed using Coomassie brilliant blue (CBB) or glycoprotein-specific (GS) staining on SDS-PAGE gels. The result showed that the bands of these purified proteins were similar on the CBB and GS staining gels (Fig. 1b), suggesting that these proteins purified using plant lectin affinity could be glycoproteins.

### Identification of the proteins enriched by lectin affinity

These enriched proteins were digested with the trypsins and the peptide mixtures were analyzed by LC-MS/MS. Based on the annotation of the Populus protein database, the peptides were further identified and assembled into protein identifications (Supplemental Table S1). A total of 154 proteins were identified by MS and database retrieval (Table 1). Among them, 36 proteins were matched with a peptide sequence, and the remaining coincided with more than two peptides. In addition, the peptide number, the score, and coverage rate for each identified protein are shown in Table 1.

**Table 1:** Identification of the proteins enriched by lectin affinity from poplar developing xylem.

Protein name	Gi number	Phytozome accession no.	Peptides	Score	Cov %	N-/O-linked sites
**Proteins acting on polysaccharides (47)**						
GH3 Beta-xylosidase	222845455	Potri.001G206800	9	432	16	Y/Y
GH3 Beta-xylosidase	222846715	Potri.001G089100	6	188	10	Y/Y
GH3 Beta-xylosidase	222861083	Potri.014G122200	6	285	11	Y/N
GH3 Beta-xylosidase	222844484	Potri.002G197200	1	76	1	Y/N
GH3 Beta-glucosidase	222852772	Potri.007G114300	1	48	1	Y/Y
GH5 Mannan endo-1,4-beta-mannosidase	222855167	Potri.006G109900	2	86	6	Y/Y
GH16 Xyloglucan endo-transglycosylase	118481141	Potri.003G159700	6	483	26	Y/N
GH16 Xyloglucan endo-transglycosylase	124109187	Potri.001G071000	6	414	28	Y/N
GH16 Xyloglucan endo-transglycosylase	222857312	Potri.013G005700	2	203	9	Y/Y
GH17 Glucan endo-1,3-beta-glucosidase	222850378	Potri.009G076500	3	86	8	Y/Y
GH17 Glucan endo-1,3-beta-glucosidase	222858075	Potri.013G059700	12	1333	39	Y/N
GH17 Glucan endo-1,3-beta-glucosidase	222862285	Potri.019G032900	10	1131	29	Y/Y
GH17 Glucan endo-1,3-beta-glucosidase	222873604	Potri.018G150400	7	671	20	Y/Y
GH 27 Alpha-galactosidase	222862356	Potri.019G056700	1	64	2	Y/Y
GH28 polygalacturonase-like	222843096	Potri.002G162400	5	446	20	Y/N
GH28 polygalacturonase	222863392	Potri.010G005500	3	167	9	Y/N
GH28 polygalacturonase	222838571	Potri.008G211500	3	160	10	Y/N
GH28 polygalacturonase	222861707	Potri.014G112100	2	134	7	Y/N
GH28 polygalacturonase	222837934	Potri.003G131700	1	125	3	Y/Y
GH28 polygalacturonase	222860156	Potri.011G159000	2	84	4	Y/N
GH28 polygalacturonase	222843280	Potri.002G186900	2	60	6	Y/N
GH28 polygalacturonase	222867323	Potri.016G051200	2	53	4	Y/Y
GH31 Glucan 1,3-alpha-glucosidase	222853440	Potri.007G100000	10	287	13	Y/N
GH31 Glucan 1,3-alpha-glucosidase	222856503	Potri.005G069000	4	97	6	Y/Y
GH32 beta-fructofuranosidase	222868827	Potri.015G127100	2	46	5	Y/Y
GH38 alpha-mannosidase	222843486	Potri.002G238200	14	859	19	Y/N
GH38 alpha-mannosidase	222861848	Potri.014G143600	10	521	15	Y/N
GH38 alpha-mannosidase	222859443	Potri.012G106500	5	374	7	Y/Y
GH38 alpha-mannosidase	222859442	Potri.012G106400	1	50	7	N/Y
GH51 Alpha-L-arabinofuranosidase	222853916	Potri.006G029900	2	269	5	Y/Y
GH127 Beta-L-arabinofuranosidase	222845043	Potri.001G018200	5	154	7	Y/Y
Alpha-fucosidase	222863630	Potri.010G047900	3	151	11	Y/N
Xylose isomerase	222865922	Potri.T093900	13	998	37	N/N
Pectinesterase	222861105	Potri.014G127000	2	285	6	Y/Y
Pectinesterase	222844452	Potri.002G202600	1	263	3	Y/Y
Pectin lyase	118488323	Potri.003G175900	2	71	6	Y/Y
Acetylglucosaminyl transferase	222845138	Potri.001G068100	2	48	7	Y/N
Glycopeptide N-glycosidase	222859921	Potri.011G109700	7	268	11	Y/Y
Glucosidase II beta subunit	222872983	Potri.006G061600	1	74	4	Y/N
Fasciclin-like arabinogalactan protein	222861509	Potri.014G071700	2	99	7	Y/Y
Fasciclin-like arabinogalactan protein	47717933	Potri.015G129400	2	96	10	Y/Y
Fasciclin-like arabinogalactan protein	118482997	Potri.012G127900	2	84	10	Y/Y
Non-classical arabinogalactan protein	118482413	Potri.002G093100	1	82	10	N/Y
Non-classical arabinogalactan protein	118481929	Potri.004G044700	1	79	6	Y/Y
COBRA-like protein	118485798	Potri.010G001100	5	232	12	Y/Y
COBRA-like protein	118488472	Potri.015G060100	4	145	11	Y/N
COBRA-like protein	118482010	Potri.015G060000	1	50	2	Y/Y
**Oxido-reductases (43)**						
Multicopper oxidase, SKU5-like protein	222871142	Potri.001G120300	13	1097	36	Y/Y
Multicopper oxidase, SKU5-like protein	222840952	Potri.003G112700	12	944	34	Y/Y
Multicopper oxidase, SKS1-like protein	222859558	Potri.012G126400	5	232	14	Y/Y
Multicopper oxidase, SKS1-like protein	222868828	Potri.015G127200	3	167	8	Y/Y
Multicopper oxidase, SKS4-like protein	118487967	Potri.004G010100	1	182	2	Y/N
Multicopper oxidase	222857214	Potri.005G247700	10	1031	30	Y/N
Multicopper oxidase	222842395	Potri.002G013700	14	997	27	Y/N
Multicopper oxidase	222853065	Potri.007G038300	10	844	29	Y/Y
Multicopper oxidase	118488761	Potri.001G219300	9	526	26	Y/Y
Multicopper oxidase	222849177	Potri.004G180500	12	470	26	Y/N
Multicopper oxidase	222843342	Potri.002G227600	2	194	6	Y/Y
Multicopper oxidase	222855045	Potri.006G087500	4	142	9	Y/Y
Multicopper oxidase	222844867	Potri.001G000600	1	109	2	Y/N
Multicopper oxidase	222849246	Potri.009G159700	4	56	9	Y/Y
Laccase	222852007	Potri.010G183500	6	1201	16	Y/Y
Laccase	222852006	Potri.008G073700	9	1113	23	Y/Y
Laccase	222864170	Potri.010G183500	5	1006	13	Y/Y
Laccase	222864171	Potri.010G183600	5	639	13	Y/N
Laccase	222854184	Potri.006G096900	3	95	7	Y/Y
Laccase	222849832	Potri.009G034500	3	79	7	Y/N
Laccase	222850532	Potri.009G042500	1	65	2	Y/Y
Laccase	222846554	Potri.001G054600	1	64	3	Y/N
Laccase	3805960	Potri.010G193100	1	46	2	Y/Y
peroxidase	115345276	Potri.003G214700	5	514	21	Y/N
Peroxidase	118487605	Potri.005G195600	3	55	12	Y/Y
FAD-Berberine enzyme 545aa	222860154	Potri.011G158700	7	323	15	Y/Y
FAD-Berberine enzyme	222846288	Potri.001G462800	5	298	10	Y/N
FAD-Berberine enzyme	222833370	Potri.006G128900	3	223	5	Y/N
FAD-Berberine enzyme	222872123	Potri.011G159600	2	175	3	Y/N
FAD-Berberine enzyme	222846286	Potri.001G462600	2	175	2	Y/Y
FAD-Berberine enzyme	222858409	Potri.012G034700	2	175	3	Y/N
FAD-Berberine enzyme	222834675	Potri.011G160300	2	173	5	Y/N
FAD-Berberine enzyme	222872175	Potri.011G161500	2	89	4	Y/N
FAD-Berberine enzyme	222846302	Potri.001G464700	1	83	2	Y/Y
FAD-Berberine enzyme	222872118	Potri.011G161400	1	80	2	Y/N
FAD-Berberine enzyme	222847838	Potri.001G470100	1	80	2	N/Y
FAD-Berberine enzyme	222860155	Potri.011G158800	1	80	2	Y/Y
Protein disulfide isomerase	222842706	Potri.002G082100	23	3116	50	Y/Y
Protein disulfide isomerase	118485031	Potri.009G013600	14	648	29	Y/N
Protein disulfide isomerase	222846968	Potri.001G183500	2	55	5	Y/Y
Chitinase-like	118481023	Potri.010G141600	2	65	9	Y/Y
Cytochrome P450	222868639	Potri.015G085800	1	56		N/Y
Cu/Zn superoxide dismutase	4102861	Potri.005G044400	1	63	10	Y/Y
**Proteases (26)**						
Aspartyl protease	118482048	Potri.001G028200	5	244	15	Y/Y
Aspartyl protease 439aa	222847473	Potri.001G306200	1	90	3	Y/N
Serine carboxypeptidase	222849960	Potri.009G003100	2	129	7	Y/N
Serine carboxypeptidase	222850469	Potri.009G055900	4	88	8	Y/Y
Serine carboxypeptidase S28	222854432	Potri.006G207900	3	499	10	Y/Y
Serine carboxypeptidase S28	222836225	Potri.007G015400	6	428	14	Y/N
Serine carboxypeptidase S28	118487876	Potri.007G015300	5	418	13	Y/N
Serine carboxypeptidase S28	222853228	Potri.007G008100	4	224	12	Y/N
Subtilase family protein	222860749	Potri.011G155400	4	269	7	Y/N
Subtilase family protein	222875305	Potri.001G440300	5	243	9	Y/N
Subtilase family protein	222848475	Potri.004G173900	1	81	1	Y/Y
Subtilase family protein	222854095	Potri.006G076200	3	76	5	Y/Y
Peptidase M20/M25/M40	222863686	Potri.010G076100	11	1315	33	Y/N
Peptidase M20/M25/M40	118486005	Potri.009G169300	12	912	41	N/Y
Peptidase M20/M25/M40	222837797	Potri.004G208100	4	526	12	N/Y
Peptidase M20/M25/M40	222842722	Potri.002G085400	5	205	16	Y/Y
Peptidase M20/M25/M40	222840651	Potri.003G045200	2	131	5	Y/N
Peptidase M28 family	222855209	Potri.006G153300	4	178	10	Y/N
Cysteine proteinase	222856445	Potri.005G256000	4	320	16	Y/N
Cysteine proteinase	222843627	Potri.002G005700	4	223	21	Y/N
Cysteine proteinase	222837653	Potri.004G207600	3	212	12	Y/N
Cysteine proteinase	118482340	Potri.006G141700	1	59	3	Y/N
Proteinase inhibitor	118485178	Potri.013G112800	3	56	39	N/Y
Proteinase inhibitor	118482991	Potri.019G083300	2	51	20	N/Y
Amidohydrolase family	222849678	Potri.009G067700	5	122	12	Y/Y
Amidohydrolase family	222847228	Potri.001G273400	4	59	11	Y/Y
**Protein kinase (8)**						
LRR protein kinase	222868332	Potri.016G144100	4	480	7	Y/Y
LRR protein kinase	222853199	Potri.007G014700	3	231	4	Y/Y
LRR protein kinase	222863806	Potri.010G103000	4	133	7	Y/Y
LRR protein kinase	222854082	Potri.006G073900	4	110	6	Y/Y
LRR protein kinase	222866571	Potri.018G107400	2	98	2	Y/Y
LRR protein kinase	222852307	Potri.008G140500	1	45	1	Y/Y
LRR protein kinase	222856570	Potri.005G083000	3	45	3	Y/Y
LRR protein kinase	222852450	Potri.008G176900	1	47	2	Y/N
**Proteins with interacting/binding domains (9)**						
Leucine-rich repeat protein	190897432	Potri.009G064300	9	663	42	Y/N
Leucine-rich repeat protein	222853264	Potri.007G001000	5	348	12	Y/N
Leucine-rich repeat protein	222854117	Potri.018G151000	2	132	4	Y/Y
Leucine-rich repeat protein	222846498	Potri.001G017500	1	46	2	Y/N
HSP70 family protein	222867185	Potri.016G019800	13	998	24	Y/N
HSP70 family protein	222854802	Potri.006G022100	14	939	20	Y/Y
HSP70 family protein	222841104	Potri.003G143600	1	62	2	N/Y
Calreticulin family protein	222871704	Potri.005G015100	11	834	34	Y/Y
Calreticulin family protein	118485765	Potri.013G009500	10	653	38	Y/Y
**Proteins related to lipid metabolism(6)**						
Lipase/lipooxygenase	118483838	Potri.005G076900	5	287	40	Y/N
Purple acid phosphatase	222865126	Potri.010G158400	3	119	7	Y/N
Purple acid phosphatase	222851161	Potri.008G096000	3	109	6	Y/N
HAD superfamily protein	222839124	Potri.004G232900	2	63	9	Y/N
Type I phosphodiesterase	222855200	Potri.006G150900	2	160	7	Y/Y
Type I phosphodiesterase	222872448	Potri.018G066600	1	79	3	Y/Y
**Amino acid metabolism (4)**						
Amidase family protein	222869309	Potri.015G109400	4	180	12	Y/Y
Methionine synthase	222850043	Potri.009G152800	2	56	4	Y/Y
Methionine synthase	118483919	Potri.013G061800	2	56	4	Y/Y
Cysteine desulfurase	222850426	Potri.009G066000	1	55		Y/Y
**Miscellaneous proteins (6)**						
Germin-like protein 10	118482567	Potri.002G184900	4	623	19	Y/Y
Cyclase family protein	222850275	Potri.009G097300	2	192	13	Y/N
Cyclase family protein	118488222	Potri.001G301600	4	95	18	Y/Y
Kelch repeat protein	222845394	Potri.001G178500	1	73		Y/N
Nucleosome assembly protein	222854259	Potri.006G148600	1	56	3	Y/N
Cupin domain protein	222858047	Potri.013G051600	1	55	7	Y/N
**Unknown function (5)**						
Unknown protein (Duf642)	118486479	Potri.011G087500	4	124	16	Y/Y
Unknown protein (Duf568)	118487890	Potri.002G249200	1	54	5	Y/Y
Unknown protein (Duf2828)	222850304	Potri.009G091400	1	57		Y/Y
Unknown protein	222846617	Potri.001G068800	1	80	6	Y/Y
Unknown protein	118486279	Potri.019G076900	2	68	5	Y/Y

### Glycosylation site analysis of the identified glycoproteins

N- and/or O-glycosylation sites of the identified proteins were analyzed by NetNGlyc, NetOGlyc and GlycoEP tools, and the results are shown in Supplemental Table S2. Of all 154 proteins, 153 proteins contained N- and/or O-glycosylation sites and only one did not contain the glycosylation sites. Among them, 143 proteins had the N-glycosylation sites; 56 proteins contained 1−3 N-glycosylation sites, and 41 and 46 proteins with 4−6 and 7~ N-glycosylation sites, respectively (Supplemental Fig. S1a). However, O-glycosylation of protein and its types are more complex in plants and the current tools are difficult to accurately predict O-glycosylation sites in plant proteins. The analysis suggested that the 56 proteins might contain 1−3 O-glycosylation sites, and 17 and 19 proteins with 4−6 and 7~ O-glycosylation sites, respectively (Supplemental Fig. S1b).

**Figure 2:**
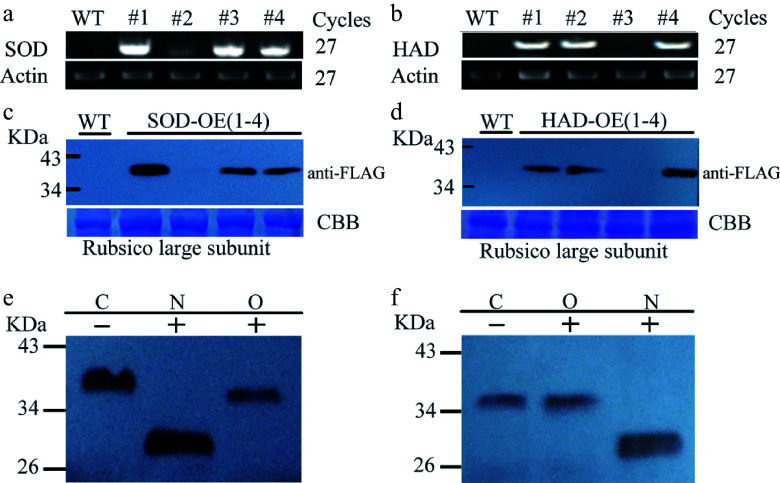
Verification of N- and/or O-glycosylation in PtSOD and HAD proteins. (a), (b) Analysis of PtSOD or PtHAD gene expression by RT-PCR in their transgenic Arabidopsis plants. AtActin2 serves as a control gene. (c), (d) Western blot analysis of PtSOD1-Flag and HAD-Flag protein levels in corresponding transgenic plants usig anti-FLAG antibody. Coomassie brilliant-stained Rubsico large subunit proteins indicate the loading amount of each sample on SDS-PAGE gels as control. (e), (f) Migration analysis of PtSOD and PtHAD proteins on SDS-PAGE gels. Protein extracts with/without the digestion of PNGase F (N) or O-glycosidase (O) were separated on 10% SDS-PAGE gels and followed by immunoblotting with anti-FLAG antibody.

To confirm whether the N/O-glycosylation prediction of the identified proteins is reliable, we selected two proteins with less glycosylation sites predicted by the tools for verification. A selected superoxide dismutase (PtSOD) contained two N- and one O-glycosylation sites, while the HAD (PtHAD) only contained two N- glycosylation sites (Supplemental Fig. S1). We first generated PtSOD- and PtHAD-transgenic Arabidopsis plants, respectively. RT-PCR analysis showed that the three transgenic lines overexpressed *PtSOD* and *PtHAD* genes, respectively (Fig. 2a, b). Because of the expressed PtSOD or PtHAD with the fusion of FLAG tag, we further detected PtSOD or PtHAD protein level in transgenic lines by Western blot using anti-FLAG antibody. The data showed high PtSOD or PtHAD protein levels in transgenic lines (Fig. 2c, d). In addition, Western blot analysis revealed that the molecular weights (MWs) of the expressed PtSOD and PtHAD proteins were 38 and 37 kDa, respectively (Fig. 2d). The MWs are much bigger than those calculated based on the amino acids of PtSOD or PtHAD proteins, suggesting that protein modification might occur. Next, protein extracts from the PtSOD- or PtHAD-transgenic plants were digested by O-glycosidase and PNGase F, respectively. Western blot analysis showed that the digestion of O-glycosidase or PNGase F accelerated the migration rate of PtSOD on SDS-PAGE gels (Fig. 2e), indicating that the PtSOD protein is both N- and O-glycosylated. The migration of PtHAD digested by PNGase F was accelerated on the SDS-PAGE gel, while that of PtHAD digested by O-glycosidase did not change (Fig. 2f). This suggests that PtHAD protein has N-glycosylation sites and no O-glycosylation sites. Taken together, this data indicates that analysis of N- and/or O-glycosylation sites of the identified proteins by the bioinformatic tools is reliable.

### Functional classification and localization of the identified glycoproteins

**Figure 3:**
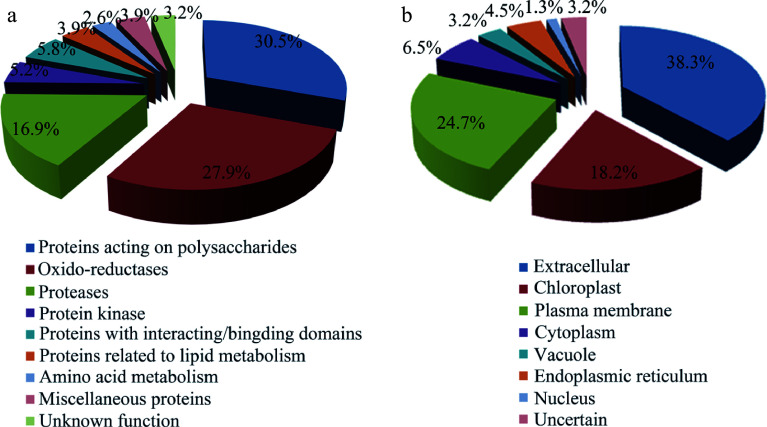
Functional classification and localization of the identified glycoproteins. (a) Functional classification of glycoproteins identified from Populus developing xylem. Please refer to Supplemental Table S1 for detailed analysis. (b) Subcellular localization of the identified glycoproteins predicted by Plant-mPLoc, ngLOC, ProtComp9.0, WoLF PSORT and YLOC.

The identified glycoproteins were classified into nine functional groups based on gene annotations and/or known domains (Fig. 3a), which include protein acting on carbohydrates (30.5%), oxido-reductase (27.9%), proteases (16.9%), protein kinases (5.8%), proteins with interaction domain (5.8%), lipid metabolism (3.9%) and amino acid metabolism (2.6%). The group of proteins acting on carbohydrates mainly includes beta-xylosidase (GH3), xyloglucan endo-transglycosylase (GH16), glucan endo-1,3-beta-glucosidase (GH17), polygalacturonase (GH28), alpha-L-arabinofuranosidase (GH51) and fasciclin-like arabinogalactan protein (FLA). Oxido-reductase cluster contained multicopper oxidases, laccases, FAD-berberine enzymes and peroxidases. Protease is the third largest cluster of the identified glycoproteins, including aspartyl proteases, serine carboxypeptidases, subtilases, peptidases, cysteine proteinases. LRR protein kinases and leucine-rich repeat proteins were also important functional groups of the identified glycoproteins.

Subcellular localization of the identified glycoproteins was analyzed using five bioinformatic tools including Plant-mPLoc, ngLOC, ProtComp 9.0, WoLF PSORT and YLOC. As shown in Fig. 3b, the 38.3% of glycoproteins identified were extracellular proteins (cell wall protein), and the 24.7 and 18.2% of the identified glycoproteins were located in the plasma membrane and chloroplast, respectively. In addition, a small proportion of glycoproteins were located in cytoplasm (6.5%), endoplasmic reticulum (4.5%), vacuole (3.2%), or nucleus (1.3%). Most glycoproteins were secreted proteins, which contain the signal sequences (Table 1).

### Expression profiles of the genes encoding the identified glycoproteins

To analyze the potential roles of these glycoproteins in developing xylem, the digital expression profiles of the genes encoding the glycoproteins were collected from poplar electronic fluorescent pictograph (eFP) browsers (Supplemental Fig. S2). Of all 154 encoding genes, the 34 genes have no corresponding data in the eFP database. The eFP data showed that profiles of 52 gene expression were high in developing xylem, and transcription levels of 21 genes were high in roots. In addition, 21 and 14 genes were highly expressed in female and male catkins, and only 11 genes in young leaves.

**Figure 4:**
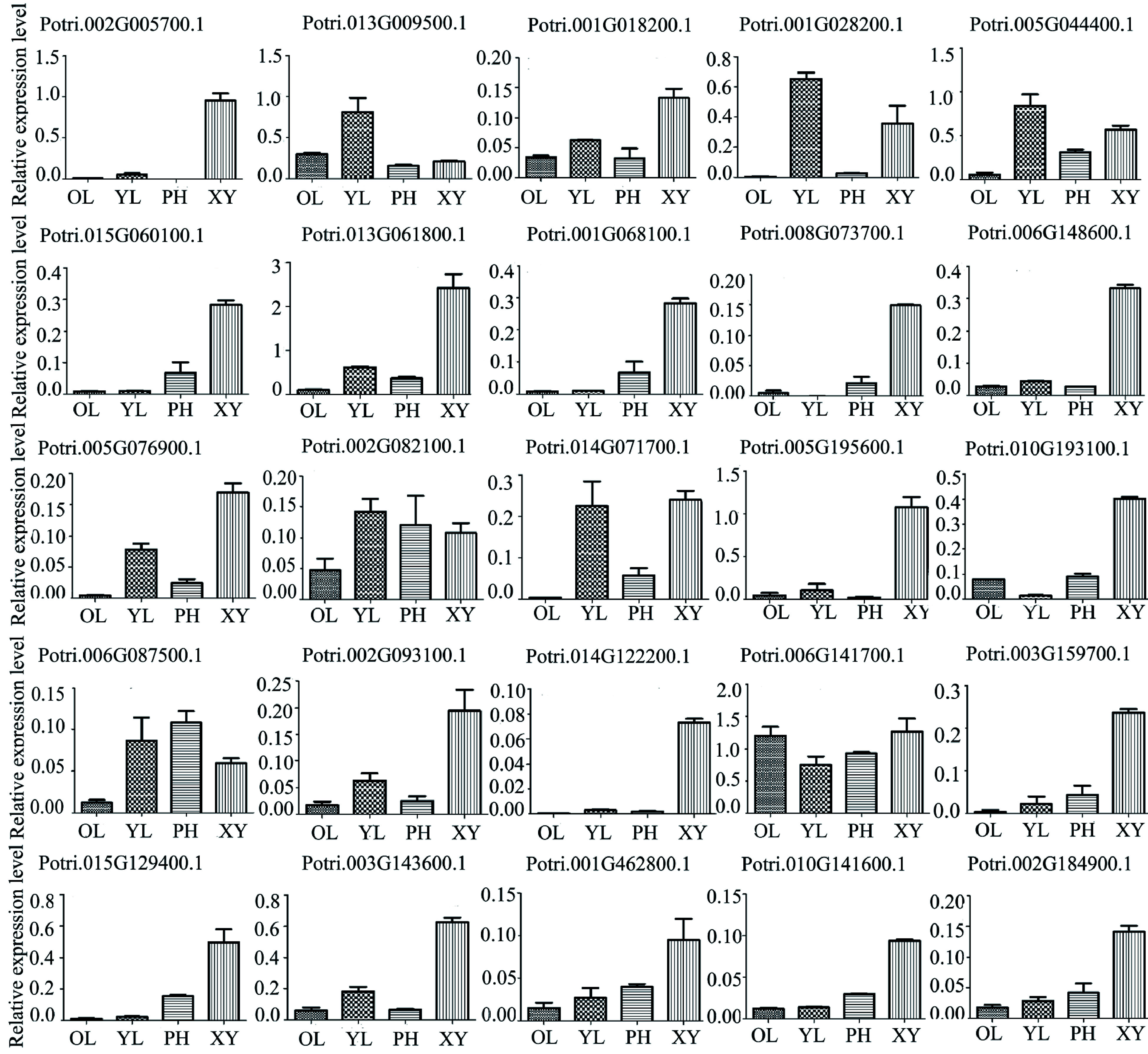
Expression profiles of 25 genes encoding the glycoproteins in different poplar tissues using RT-qPCR analysis. Different tissues included phloem (PH), xylem (XY), young leaf (YL) and mature leaf (OL). The expression of *PtActin2* was used as an internal control. Data are means ± standard error of three technical replicate results.

To test the eFP data above, we further examined expression profiles of the 52 genes using RT-qPCR. The results showed that only 25 genes were highly expressed and their expression profiles were more specific in xylem (Fig. 4). These xylem-expressed genes encode laccase, FLA, peroxidase, methionine synthase, and cysteine protease. Laccase, peroxidase and FLA play a key role in secondary cell wall (SCW) formation, and cysteine protease is involved in the process of programmed cell death (PCD) and SCW thickening^[30−33]^. These expression profiles suggest that many glycoproteins identified should be of importance for wood cell wall synthesis and modification in poplar.

## DISCUSSION

Lectin affinity enrichment is based on the specific binding interaction between lectins and unique glycan structures attached to glycoproteins. A variety of lectins can selectively bind to oligosaccharides, and enrich different types of the glycans of glycoproteins^[34−36]^. To date, most of the work using LAC for targeted glycoprotein enrichment in plants has focused on N-glycosylation, and binding specificity of the lectin for O-glycosylation is less satisfactory. To capture O-glycoproteins as far as possible, we made serial columns of concanavalin A and jacelin in tandem to isolate O-glycoproteins from the developing xylem in poplar. As a result, many O-glycoproteins were identified in the present study. In addition, some studies rely on two-dimensional electrophoresis (2-DE), which has limitations when used to separate and identify certain types of proteins, such as those that are membrane-associated, less abundant, or have extreme p*I*s or MWs^[37]^. Overall, our strategy of this study is a relatively unbiased technology that can more comprehensively identify glycoproteins.

Protein glycosylation occurs in the proteins in the secretory pathway, so a convenient indicator for evaluating the identified glycoproteins is to use software packages searching signal peptides. Up to 89% of the glycoproteins identified from poplar developing xylem were suggested to have signal peptides (Supplemental Table S3). This is much higher than other plant extracellular proteomics, based on the way that LAC is not used^[38, 39]^. Proteins with signal peptides entering the secretory pathway do not necessarily target the cell wall, but may remain on the endomembrane system, such as endoplasmic reticulum, Golgi apparatus, and other organelles, including vacuoles and chloroplasts. We analyzed the localization of identified glycoproteins using the software subcellular localization website. According to predictive analysis (Fig. 3), the proportion of proteins (38.3%) was located in cell wall, while most of the remaining proteins might be in the plasma membrane (24.7%). It provides a hint that xylem synthesis and modification might be mediated by a number of the glycoproteins in the cell wall and/or plasma membrane.

**Figure 5:**
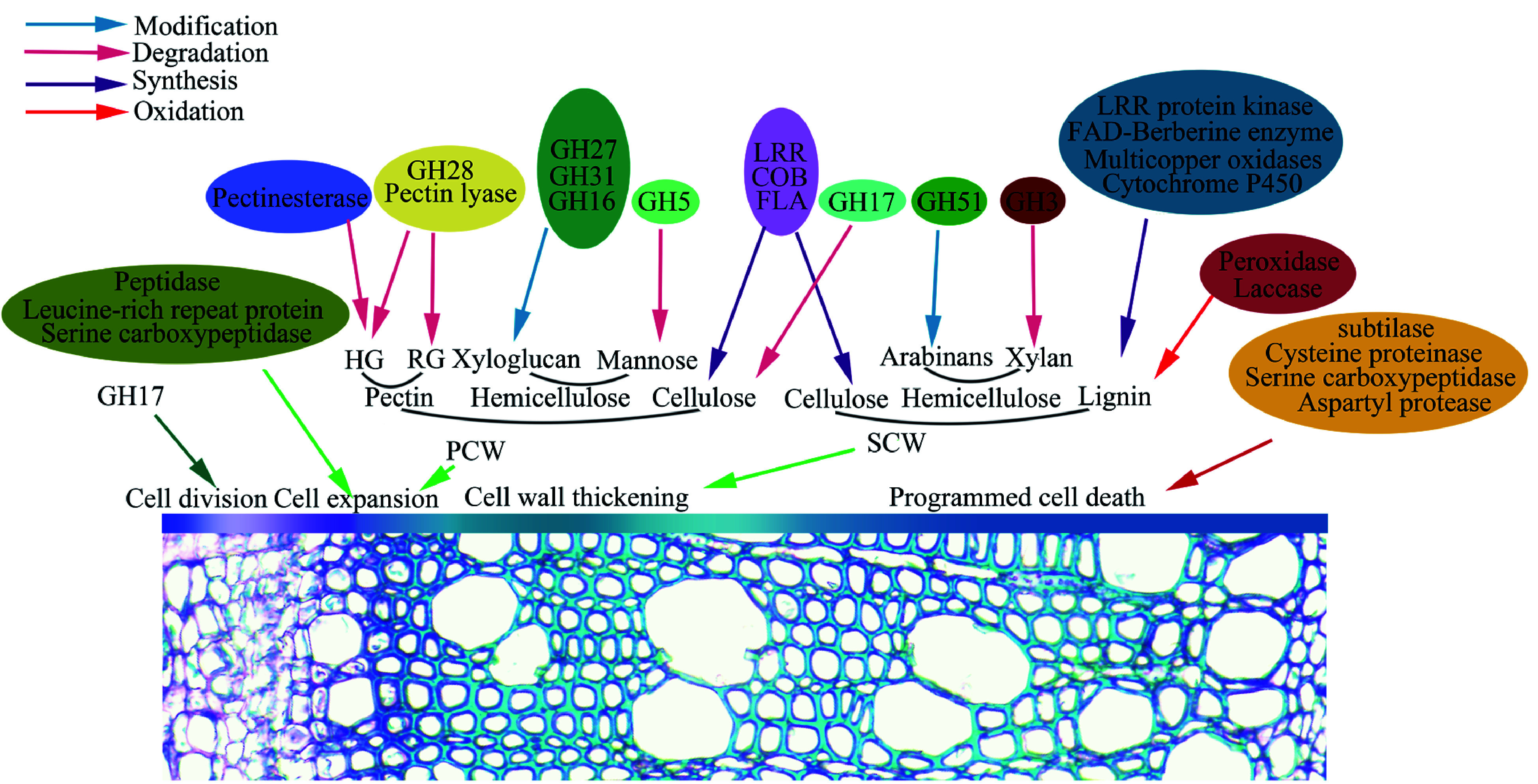
Identified glycoproteins are proposed to be involved in wood formation that includes cambia cell division, cell differentiation and expansion, SCW synthesis and PCD.

In this study, most of the identified glycoproteins clustered a functional group of protein acting on carbohydrates (Table 1), suggesting their involvement in wood formation in poplar. Wood formation undergoes a genetically controlled xylogenesis process, which includes cambia cell division, cell differentiation and expansion, SCW synthesis and PCD. As shown in Fig. 5, a number of the glycoproteins identified are involved in wood formation. The PCWs of the growing xylem cells are mainly composed of pectins (such as rhamnogalacturons and homogalacturonans), cellulose, and hemicellulose (xylogucan and mannan). Here we have identified 33 GHs in poplar secondary xylem (Table 1), which belong to 11 types of glycoside hydrolases (GHs). GHs are important cell wall polysaccharide-modified enzymes that participate in the division and expansion of plant cells and their substrates are pectin and hemicellulose^[40−46]^. GH16, GH31 and GH51 may act on modification of xylans in cell wall, and GH28 can hydrolyze pectin^[47, 48]^. GH38 may be involved in the modification of the mannose and GH32 as the invertase functions in carbohydrate allocation^[49−51]^. GH3 and GH5 have broader substrates. It is reported that they are involved in modification and hydrolysis of hemicellulose, as well as lignification and secondary growth^[41,45]^. In addition to GHs, pectin modifying enzymes, pectin esterase and pectin lyase, affect the plasticity and fluidity of cell walls and play a decisive role in the final shape and size of cells^[52, 53]^. In addition, peptidase and serine carboxypeptidase affect cell expansion, but the mechanism is unknown^[54]^.

When xylem cells reach the final size and shape, a thicker SCW is produced continuously. In this study, dozens of glycoproteins including laccase, peroxidase and methionine synthase, are specifically expressed in secondary xylem at the transcriptional level, suggested by the RT-PCR analysis (Fig. 4). Lignin is one of main components of secondary xylem (wood) in trees. In this study, 38 glycoproteins identified might be involved in lignin biosynthesis, which include BBE, laccase and peroxidase (Table 1). BBE-like proteins, as monolignol oxidoreductases, may participate in the oxidation of lignin required for polymerization processes, while laccase and peroxidase are responsible for the polymerization of the lignins^[55−58]^. Other glycoproteins, such as FLAs, COBRA-like protein and LRR protein kinase participate in cell wall thickening, for example, cellulose deposition in the SCW is implemented by the COBRA-like protein^[59−62]^. Additionally, we also identified several types of proteases such aspartyl protease, serine carboxypeptidase and cysteine proteinase, which are involed in cell death of xylem fibers and vessels^[63−65]^. Overall, these glycoproteins could be served or proposed as the players in wood formation in poplar. Recently, poplar mannanase PtrMAN6 with the N-glycosylation plays a role in coordinating cell wall remodeling with suppression of secondary wall thickening^[66]^. Another study reveals that glycan synthesis levels of the AGP proteins change in wood formation^[67]^. Thus, it is inferred that protein glycosylation as a regulatory way should be involved in wood formation. We are now attempting to detect the roles of glycosylation sites of the glycoprotein in this process through genetic studies.

## MATERIALS AND METHODS

### Plant material and growth conditions

In this study, we used *Populus simonii* ×* P. nigra* as plant material. Three-year-old trees grown in a forest farm at Northeast Forestry University (Harbin, China, longitude 127°18′0″; latitude 45°2′20″) were selected for correcting developing xylem tissues. Arabidopsis thaliana (Columbia ecotype) plants were grown in the greenhouse (16 h light/8 h dark) at a light intensity of 120 μmol photons m^− 2^ s^− 1^ at 22 °C. The CDS of PtHAD or PtSOD was amplified using the xylem cDNAs as a template, and the DNA fragements were constructed into pGWB11 vector with the fusion of FLAG tag for overexpression of PtHAD or PtSOD. After DNA sequencing, the resultant constructs were introduced into *A. tumefaciens* strain GV3101 for Arabidopsis transformation using the floral-dip method.

### Protein extraction

Developing xylem tissue was corrected from one young tree on June 15, and we repeated it three times. After the bark was peeled, the xylem tissue was quickly frozen with liquid nitrogen and developing xylem cells were scraped from the outside to the inside. Three corrected sample materials were mixed and ground into a powder. Approximately 50 g powder was saturated into 120 ml protein extraction buffer (50 mM Tris-HCl, pH 7.4, 150 mM NaCl, 1 mM MgCl_2_, 1 mM CaCl_2_, 1 mM MnCl_2_). After the mixture was shaken on ice for 30 min, the homogenate was centrifuged at 40,000 g for 30 min at 4 °C. The supernatant was used for enrichment of glycoproteins using lectin affinity chromatography.

### Lectin affinity chromatography

The crude protein was used for lectin affinity chromatography. Four plant lectins, concanavalin A (Con A), Triticum vulgaris (WGA), peanut (PNA) and jacalin, are used to specifically enrich different sugar residues of various glycoproteins. We added 0.5 ml ConA-Sepharose 4B (27700, Supelco), WGA-Agarose (L1882, Sigma-Aldrich), PNA-Agarose (AL-1073, Vector Laboratories), and Jacalin-Sepharose (6561, Biovision) to the columns, respectively. After 5 ml binding buffer (20 mM Tris-HCl, pH 7.4, 150 mM NaCl, 1 mM MgCl_2_, 1 mM CaCl_2_, 1 mM MnCl_2_) was added to clean the column, the crude protein in the supernatant was successively passed through the four columns. Then, each column was washed with 50 ml of binding buffer to remove the unbound proteins. After discarding the binding buffer, the bound glycoproteins were respectively eluted from the four columns with 0.5 ml of elution buffer (20 mM Tris-HCL, pH 7.4, 300 mM NaCl, 1 mM MgCl_2_, 1 mM CaCl_2_, 1 mM MnCl_2_) containing 500 mM methyl α-D-glucopyranoside (M9376, Sigma-Aldrich) for ConA, 500 mM N-Acetyl-D-glucosamine (A8625, Sigma-Aldrich) for WGA, 500 mM N-Acetyl-D-galactosamine (A2795, Sigma-Aldrich) for PNA, or 500 mM galactose (G0750, Sigma-Aldrich) for jacalin. The eluted fractions of the sample were pooled and filtered through Microcon YM-10 centrifugal filter devices to a volume of ~0.2 ml. The sample was used for protein identification.

### Protein identification and database searching

The sample was digested with porcine trypsin (Promega) at 37 °C overnight, as described previously^[68]^. After protein digestion of trypsin, the short peptides obtained were performed for LC-MS/MS analysis, as described previously^[69]^. The MS/MS spectra were searched against the NCBInr protein databases and phytozome databases using Mascot software (Matrix Sciences, UK). The search criteria included a mass accuracy of 0.3 Da, with one missed cleavage allowed, carbamidomethylation of cysteine as a fixed modification, and oxidation of methionine as a variable modification. A highly confident protein identification met the following criteria: (a) top hits in the database searching report; (b) a probability-based MOWSE score of greater than 55 (*p* > 0.01); (c) more than two peptides matched with a nearly complete y-ion series and complementary b-ion series present. Based on the MASCOT probability analysis, the significant hits were accepted as the identification of each protein.

### Microarray data, RNA isolation and RT-qPCR analyses

Tissue-specific expression data were downloaded from poplar eFP browser. The heat map was generated by Heat map illustrator (HemI) with the default settings. Total RNA was extracted from plant tissues using plant RNA Extraction Kit (Bio-Flux, China). For each sample, 1 μg of total RNA was reverse-transcribed into total cDNAs using the PrimeScript RT reagent Kit (TaKaRa, China). The qRT-PCR experiments were performed with SYBR Green (TaKaRa, China) in the ABI Prism 7500 system (Applied Biosystems, USA) according to the manufacturer's instructions. The reaction mixture (20 μl) consisted of 10 μl 2× TB Green Premix Ex Taq II (Tli RNaseH Plus), 0.8 μl of each gene-specific primer, 0.4 μl ROX Reference Dye II, 1 μl cDNA template and 7 μl distilled deionized H_2_O. The PCR parameters as follows: 95 °C for 30 s; 40 cycles of 95 °C for 5 s, 60 °C for 15 s, 72 °C for 30 s. PtActin2 was used as an internal control and the comparative Ct (2^−ΔCᴛ^) method was used to calculate gene expression levels. Three technical replicates were carried out for each sample.

### Deglycosylation experiment and Western blot

Transgenic plant materials were ground in liquid nitrogen and homogenized in protein extraction buffer (50 mM Tris-HCl, 200 mM NaCl, 2% SDS, 5 mM DTT, pH 8.0). The suspensions were centrifuged at 18,000 g for 5 min, and the supernatant (protein extract) was used for protein deglycosylation with/without PNGase F and/or O-glycosidase (New England Biolabs, UK). The treated protein extract was resolved in 10% SDS-PAGE gel and transferred into a PVDF membrane. Western blotting was performed using anti-FLAG antibody (Abmart, China) and Pierce ECL chemiluminescent Substrate (Thermo, USA).

### Bioinformatics analysis

TProtein sublocalization was predicted based on five bioinformatics tools including Plant-mPLoc, ngLOC, ProtComp 9.0, WoLF PSORT and YLOC. Signal peptides were analyzed using the SignalP 4.1 Server (www.cbs.dtu.dk/services/SignalP). Glycosylation sites were analyzed using three tools including NetNGlyc 1.0, NetOGlyc 4.0 and GlycoEP.

## SUPPLEMENTARY DATA

Supplementary data to this article can be found online.
