# Supplementary figures and images for: Lectin affinity-based glycoproteome analysis of the developing xylem in poplar
Source: For Res (Fayettev). 2022 Oct 27;2:13. doi: 10.48130/FR-2022-0013 (PMC11524310; doi:10.48130/FR-2022-0013)

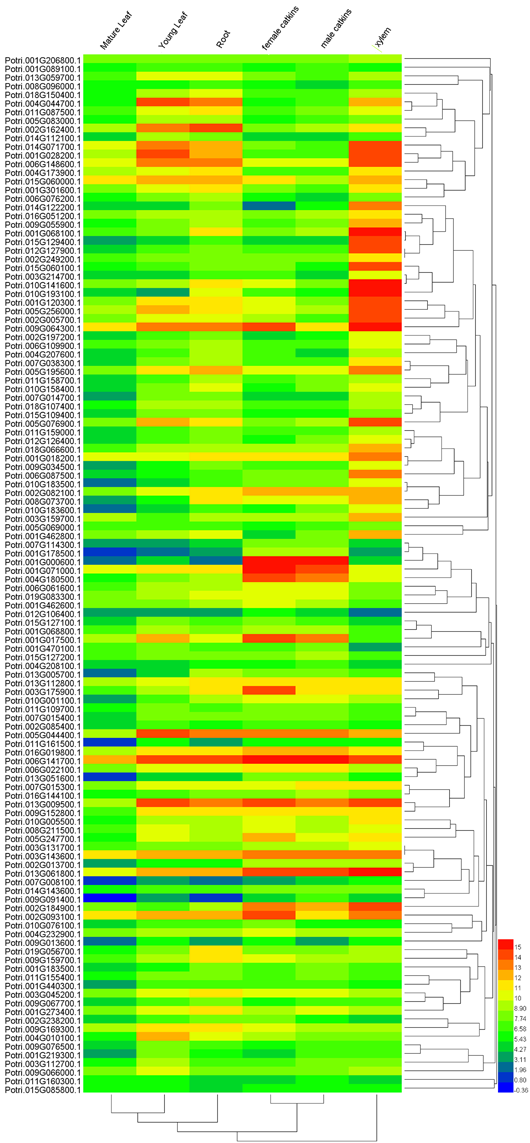

Supplement: Supplementary file 1 — Supplementary data to this article can be found online. [file FR-2022-0013-S1.zip › 10.48130_FR-2022-0013-Suppl-FigureS2.tif]

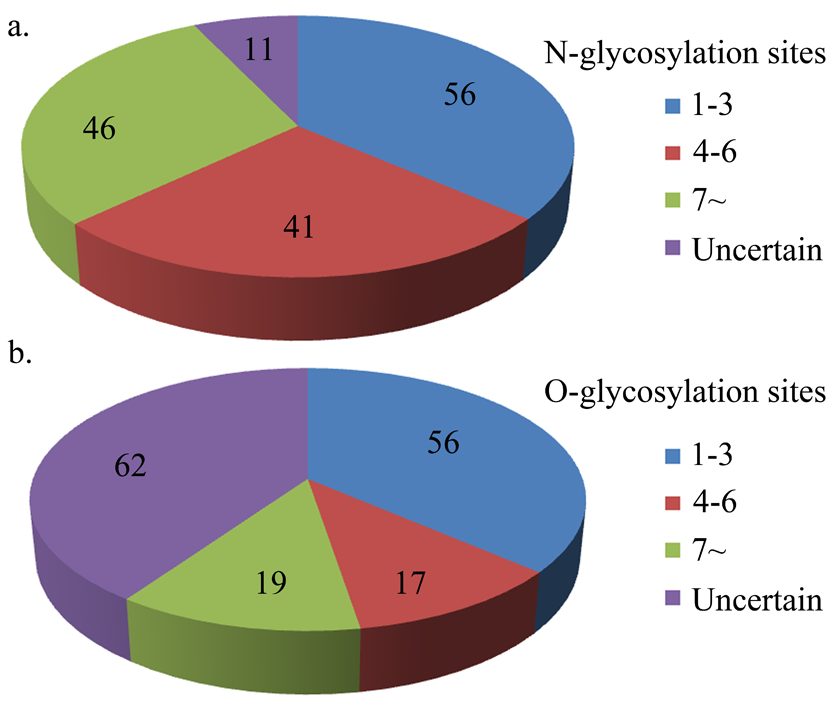

Supplement: Supplementary file 1 — Supplementary data to this article can be found online. [file FR-2022-0013-S1.zip › 10.48130_FR-2022-0013-Suppl-FigureS1.tif]
